# Estimated Human and Economic Burden of Four Major Adult Vaccine-Preventable Diseases in the United States, 2013

**DOI:** 10.1007/s10935-015-0394-3

**Published:** 2015-06-02

**Authors:** John M. McLaughlin, Justin J. McGinnis, Litjen Tan, Annette Mercatante, Joseph Fortuna

**Affiliations:** HEOR and Epidemiology, US Medical Affairs, Pfizer Inc, New York, NY USA; Schaeffer Center for Health Policy and Economics, University of Southern California, Los Angeles, CA USA; Immunization Action Coalition, St. Paul, MN USA; St. Clair County Health Department, Port Huron, MI USA; Michigan Primary Care Consortium, Lansing, MI USA; PO BOX 113, Powell, OH 43065 USA

**Keywords:** Vaccination, Immunization, Adults, Cost, Model

## Abstract

Low uptake of routinely recommended adult immunizations is a public health concern. Using data from the peer-reviewed literature, government disease-surveillance programs, and the US Census, we developed a customizable model to estimate human and economic burden caused by four major adult vaccine-preventable diseases (VPD) in 2013 in the United States, and for each US state individually. To estimate the number of cases for each adult VPD for a given population, we multiplied age-specific incidence rates obtained from the literature by age-specific 2013 Census population data. We then multiplied the estimated number of cases for a given population by age-specific, estimated medical and indirect (non-medical) costs per case. Adult VPDs examined were: (1) influenza, (2) pneumococcal disease (both invasive disease and pneumonia), (3) herpes zoster (shingles), and (4) pertussis (whooping cough). Sensitivity analyses simulated the impact of various epidemiological scenarios on the total estimated economic burden. Estimated US annual cost for the four adult VPDs was $26.5 billion (B) among adults aged 50 years and older, $15.3B (58 %) of which was attributable to those 65 and older. Among adults 50 and older, influenza, pneumococcal disease, herpes zoster, and pertussis made up $16.0B (60 %), $5.1B (19 %), $5.0B (19 %), and $0.4B (2 %) of the cost, respectively. Among those 65 and older, they made up $8.3B (54 %), $3.8B (25 %), $3.0B (20 %), and 0.2B (1 %) of the cost, respectively. Most (80–85 %) pneumococcal costs stemmed from nonbacteremic pneumococcal pneumonia (NPP). Cost attributable to adult VPD in the United States is substantial. Broadening adult immunization efforts beyond influenza only may help reduce the economic burden of adult VPD, and a pneumococcal vaccination effort, primarily focused on reducing NPP, may constitute a logical starting place. Sensitivity analyses revealed that a pandemic influenza season or change in size of the US elderly population could increase these costs dramatically.

## Introduction

Health care in the United States is facing serious challenges: access and affordability issues, discordant reimbursement systems, unnavigable health care delivery, complex reform, and underutilization of prevention, to name only a few. Although it is intuitive to scrutinize direct delivery systems and individual health outcomes to look for solutions, the use of population health analysis generally provides the best perspective on where the greatest impact with health care reform can be achieved. In a health care environment of limited resources and a rapidly aging population, it is critical to begin exploring ways to obtain data about public health interventions to ensure appropriate stewardship of scarce health care dollars. According to the Centers for Disease Control and Prevention (CDC), low uptake of routinely recommended adult immunizations remains one area of public health practice that has been insufficiently addressed at local, state, and national levels (CDC, 2012a). We determined the human and economic cost of four major vaccine-preventable diseases (VPD) among adults aged 50 years and older in the United States, namely (1) influenza, (2) pneumococcal disease, (3) herpes zoster (shingles), and (4) pertussis (whooping cough).

Influenza (flu) is a contagious respiratory illness caused by influenza viruses. The best way to prevent influenza is by getting vaccinated each year per recommendation by CDC Advisory Committee on Immunization Practices (ACIP; Grohskopf et al., 2014). Recent CDC data (2013–2014 season) suggest that the proportion of adults who were immunized against influenza was 45.3 and 65.0 % for those aged 50–64 and 65 and older, respectively (CDC, 2014).

Pneumococcal disease is an infection caused by the *Streptococcus pneumoniae* bacterium (pneumococcus). Pneumonia with empyema and/or bacteremia, febrile bacteremia, and meningitis are the most common manifestations of invasive pneumococcal disease (IPD). Nonbacteremic pneumococcal pneumonia (NPP), middle-ear infections, sinusitis, and bronchitis are non-invasive and less severe manifestations of pneumococcal infection, but are considerably more common than IPD. As of September 2014, ACIP now recommends that adults 65 years and older receive both 13-valent pneumococcal conjugate vaccine (PCV13) and 23-valent pneumococcal polysaccharide vaccine (PPSV23). Adults 65 and older who have never received a pneumococcal vaccination should receive PCV13 first, followed by PPSV23 6–12 months later. Adults who are already vaccinated with PPSV23 should receive PCV13 at least a year later (Tomczyk et al., 2014). PCV13 is also ACIP-recommended for adults younger than 65 who are immunocompromised (CDC, 2012c; Tomczyk et al., 2014), and PPSV23 is ACIP-recommended for adults younger than 65 who are immunocompetent but have underlying comorbidities that could increase their risk for developing pneumococcal disease (CDC, 2012c; Tomczyk et al., 2014). Most recent CDC estimates (2012) of pneumococcal vaccination coverage (ever received any pneumococcal vaccine) were 59.9 % among adults 65 and older, and only 20.0 % among adults younger than 65 who are at high risk for developing pneumococcal disease (Williams et al., 2014). Serious outcomes of both influenza and pneumococcal disease include hospitalization or death and occur most commonly among adults 65 and older, and among individuals with underlying comorbid disease.

Herpes zoster (shingles) is caused by the varicella zoster virus, which also causes chickenpox. For reasons that are not fully understood but that are likely due, in part, to immunosenescence with age, the virus can reactivate years after chickenpox, manifesting as shingles. The most common complication of herpes zoster is post-herpetic neuralgia (PHN) which causes severe and debilitating pain for weeks, months, or (in rare cases) years. PHN occurs in as many as a third of untreated adults aged 60 and older. Herpes zoster may also lead to serious complications involving the eye and, very rarely, pneumonia, hearing problems, blindness, brain inflammation (encephalitis), or death. ACIP recommends routine vaccination of all persons 60 and older with one dose of zoster vaccine (Hales et al., 2014; Harpaz et al., 2008). In 2012, according to the CDC, only 20.1 % of adults 60 and older reported receiving herpes zoster vaccination to prevent shingles (Williams et al., 2014).

Pertussis, also known as whooping cough, is a highly contagious respiratory disease caused by the bacterium *Bordetella pertussis*. Among adults, the disease usually results in symptoms that can be mistaken for bronchitis and upper respiratory tract infections. Untreated, adults can act as a reservoir for pertussis disease and infect younger children—where the disease can be fatal, especially in infants younger than 1 year old. Adults 65 and older should receive a single (booster) dose of tetanus toxoid, reduced diphtheria toxoid, and acellular pertussis (Tdap) vaccine. In addition, certain adults 65 and older who anticipate having close contact with an infant (e.g., grandparents, child-care providers, and health-care practitioners) should receive a single dose of Tdap to protect against pertussis and reduce the likelihood of transmission (CDC, 2011). In 2012, 24.9 % (44.8 % among health care personnel) of adults younger than 65 and 16.8 % (30.1 % among health care personnel) of adults aged 65 and older reported receiving Tdap in the last 7 years—though these percentages may be underestimated as many (>50 %) adults reported that although they did receive a tetanus vaccination, they did not know what type of tetanus vaccination they received (i.e., Td vs Tdap) (Williams et al., 2014).

Thus, as described previously, each of the four adult infectious diseases selected for this analysis has (1) at least one US Food and Drug Administration (FDA)-approved vaccine available for the prevention of vaccine-type disease, and (2) broad, age-based ACIP-recommendations for use in adults. Simply stated, for the four adult VPDs chosen, vaccination represents a safe and effective medical strategy for disease prevention that remains underutilized, and more information is needed to understand the true burden of these diseases—with the ultimate goal of raising awareness about the need for improvements in prevention efforts.

## Materials and Methods

We derived burden of disease estimates for four major adult VPDs: (1) influenza, (2) pneumococcal disease (both invasive pneumococcal disease and NPP), (3) herpes zoster (shingles), and (4) pertussis (whooping cough). We obtained estimates of adult VPD incidence and associated costs from the literature (e.g., national disease surveillance programs or large US administrative claims databases) for each of the four diseases (American Lung Association, 2010; Insinga et al., 2005; Lee et al., 2004; Masseria & Krishnarajah, 2013; Nennig et al., 1996; Nichol, 2001; Pellissier et al., 2007; Tseng et al., 2011; Weycker et al., 2010). To estimate the number of cases for each adult VPD for a given population, we multiplied age-specific incidence rates obtained from the literature by age-specific population data obtained from the 2013 Census (most recent complete US census data). We then multiplied the estimated number of cases for a given population by age-specific, estimated medical and indirect (non-medical) costs per case. Medical costs represent estimated costs for diagnostic and treatment services linked to a diagnosis of one of the four adult VPDs (Lee et al., 2004; Molinari et al., 2007; Weycker et al., 2010; Yawn et al., 2007). We derived indirect cost estimates by combining work-loss data with economic productivity data, including wages, fringe benefits/supplements, and household productivity (Grosse et al., 2009; Singhal et al., 2011; Turner et al., 2006). Neither mortality costs (i.e., the value of future income lost by premature death) nor leisure time costs (i.e., the value of time spent when not working forgone by illness) were included in indirect cost calculations. Due to the acute nature of the four infectious diseases, we did not include costs (medical or indirect) that extended beyond the year of infection.

Thus, for each disease, we developed three primary estimates to populate the cost model: (1) the estimated number of cases for a given population per year, (2) the estimated medical costs of a single case (i.e., diagnostic and treatment services related to a particular diagnosis), and (3) estimated indirect costs associated with a single case. The combination of these three estimates provided the backbone for the economic model that estimated the annual burden of adult VPD from a societal perspective. The basic formula for estimating total cost based on these three measures was:

$$ \left[ {No. \;of\;persons} \right] \times \left[ {est.\;incidence\;rate} \right] \times \left[ {\left[ {\begin{array}{*{20}c} {est.\;medical\;cost } \\ {per\;case} \\ \end{array} } \right] + \left[ {\begin{array}{*{20}c} {est.\;nonmedical\;cost } \\ {per\;case} \\ \end{array} } \right]} \right] = est.\;total\;cost $$

As a final measure, we adjusted all cost estimates to 2013 US dollars using the inpatient hospital services and medical care components of the Consumer Price Index for All Urban Consumers to inflate cost estimates for hospitalizations and outpatient care, respectively, from earlier years. Table 1 shows the underlying model assumptions (with corresponding peer-reviewed references) for (1) annual incidence rates, (2) medical costs per case, and (3) indirect costs per case by disease and age group. In addition to estimates for the cost of each of the four adult VPDs for the entire United States, we derived model estimates for each US state based on state-level, age-specific population estimates obtained from the US Census.

**Table 1:** Base-case model assumptions for US incidence rates and costs by vaccine-preventable disease and age group

Disease and age groups	Number of persons (in millions)	Incidence rate (per 100,000)	Est. medical costs (per case)	Est. indirect costs (per case)^a^
Influenza^b,c^				
50–64	61.8	6600	$1280	$604
65 and older	44.7	9000	$1867	$201
Pneumococcal^d^				
Bacteremia^d^				
50–64	61.8	20	$32,204	$2707
65–74	25.2	37	$27,883	$1086
75–84	13.5	50	$24,433	$722
85 and older	6.0	64	$19,911	$652
Meningitis^d^				
50–64	61.8	1	$35,188	$2707
65–74	25.2	2	$37,199	$1086
75–84	13.5	3	$32,957	$722
85 and older	6.0	4	$21,698	$652
Inpatient *NPP* ^d^				
50–64	61.8	57	$15,943	$2187
65–74	25.2	193	$15,887	$877
75–84	13.5	566	$15,419	$583
85 and older	6.0	1056	$14,470	$528
Outpatient *NPP* ^d^				
50–64	61.8	186	$585	$1041
65–74	25.2	370	$667	$418
75–84	13.5	620	$729	$278
85 and older	6.0	907	$801	$251
Herpes zoster^e,f,g^				
50–59	43.6	470	$1079	$3106
60–69	32.8	970	$1817	$4236
70–79	18.3	1300	$2537	$2997
80 and older	11.7	1500	$2537	$2819
Pertussis^h,i^				
50–64	61.8	292	$432	$593
65 and older	44.7	464	$432	$593

All costs were adjusted to 2013 U.S. dollars. NPP is non-bacteremic pneumococcal pneumonia caused by *S. pneumoniae*. ‘NPP inpatient’ refers to cases of NPP that require hospitalization where as ‘NPP outpatient’ refers to cases of NPP that do not require hospitalization

^a^(Grosse et al., 2009)

^b^(Molinari et al., 2007)

^c^(Turner et al., 2006)

^d^(Weycker et al., 2012)

^e^(Tseng et al., 2011)

^f^(Yawn et al., 2007)

^g^(Singhal et al., 2011)

^h^(Masseria & Krishnarajah, 2013)

^i^(Lee et al., 2004)

For each of the four major adult VPDs, we presented results for two age groups: individuals aged 50 and older and those 65 and older. We chose these age groups for two main reasons. First, these age groups closely align with FDA indications and ACIP recommendations for vaccines that protect against the four major adult VPDs evaluated in this study. Secondly, these are the most commonly used age groups when discussing adult immunizations as they reflect the point during the life course where immunosenescence has typically begun. Thus, at these two age groups, incidence rates of infectious disease begin to increase markedly and vaccination becomes particularly important. We used sensitivity analyses to simulate the impact of various epidemiological scenarios on the total estimated economic burden, and constructed 95 % confidence intervals around point estimates using a Monte Carlo simulation model. Customized estimates, with varying inputs to population, incidence, and cost parameters can be obtained for nearly any population. Interested parties should contact the manuscript’s corresponding author.

## Results

**Fig. 1:**
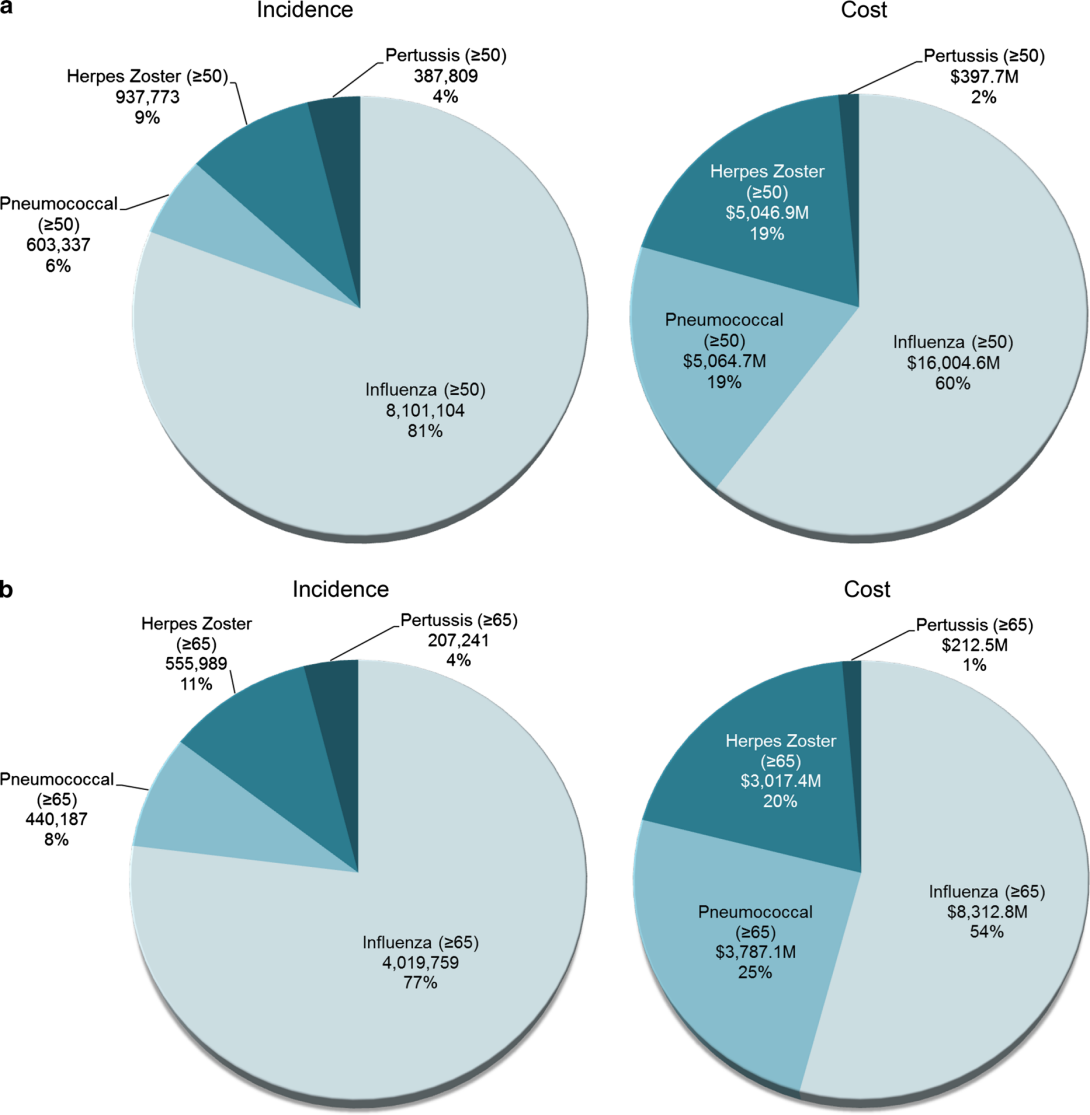
Percentage of total estimated annual number of cases and overall cost of four major adult vaccine-preventable disease in the United States, 2013 for adults aged **a** 50 years and older. **b** 65 years and older

Combining the estimated number of cases for each of the four major adult VPDs in the United States with the respective estimated medical and indirect cost per case yielded estimated total annual costs of: $16.0 billion (B) (95 % CI $9.7B, $22.0B) for influenza, $5.1B (95 % CI $4.1B, $6.4B) for pneumococcal disease, $5.0B (95 % CI $3.8B, $5.7B) for herpes zoster, and $397.7 million (M) (95 % CI $364.9M, $431.4M) for pertussis. Thus, for these four adult VPDs, the combined estimated cost burden in 2013 for US adults aged 50 and older was $26.5B (95 % CI $21.7B, $35.3B) (Table 2a). Among adults in this age group, influenza constituted 81 % of the total annual number of cases of adult VPD and 60 % of the total costs. Pneumococcal disease accounted for 6 % of total cases, but 19 % of the total costs of adult VPD. Most (80 %, $4.1B) pneumococcal costs stemmed from NPP. Herpes zoster made up 9 and 19 % of the cases and costs of adult VPD, respectively. Pertussis accounted for 4 % of total cases and 2 % of total costs of adult VPD (Fig. 1a). Among those 50 and older, medical costs accounted for 80 and 91 % of total influenza and pneumococcal costs, respectively, and 37 and 42 % of total economic burden due to herpes zoster and pertussis, respectively.

**Table 2:** Estimated annual human and economic burden of major adult vaccine-preventable disease in the United States, 2013, for adults aged, a 50 and Older, b 65 and Older

Disease	Est. cases	Est. medical cost (per case)	Est. indirect cost (per case)^a^	Est. total cost (per case)	Est. total medical cost (in millions)	Est. total indirect cost (in millions)	Est. total cost (in millions)
a							
Influenza^b,c^	8101,104	$1571	$404	$1976	$12,729.3	$3275.3	$16,004.6
Pneumococcal^d^	603,337				$4618.1	$446.5	$5064.7
Bacteremia^d^	32,041	$27,829	$1568	$29,397	$891.7	$50.2	$941.9
Meningitis^d^	2037	$33,692	$1560	$35,252	$68.6	$3.2	$71.8
NPP (inpatient)^d^	223,288	$15,335	$885	$16,220	$3424.2	$197.6	$3621.8
NPP (outpatient)^d^	345,972	$676	$565	$1241	$233.7	$195.5	$429.2
Herpes zoster^e,f,g^	937,773	$1974	$3408	$5382	$1851.0	$3195.9	$5046.9
Pertussis^h,i^	387,809	$432	$593	$1026	$167.6	$230.1	$397.7
Total	10,030,023				$19,366.1	$7147.7	$26,513.8
b							
Influenza^b,c^	4019,759	$1867	$201	$2068	$7503.3	$809.5	$8312.8
Pneumococcal^d^	440,187				$3572.2	$214.9	$3787.1
Bacteremia^d^	19,960	$25,181	$879	$26,060	$502.6	$17.6	$520.2
Meningitis^d^	1278	$32,803	$879	$33,682	$41.9	$1.1	$43.0
NPP (inpatient)^d^	187,982	$15,221	$641	$15,862	2861.3	$120.4	$2981.7
NPP (outpatient)^d^	230,968	$721	$328	$1049	$166.4	$75.8	$242.2
Herpes zoster^e,f,g^	555,989	$2354	$3074	$5427	$1308.5	$1708.9	$3017.4
Pertussis^h,i^	207,241	$432	$593	$1026	$89.6	$122.9	$212.5
Total	5223,176				$12,473.7	$2856.2	$15,329.9

All costs were adjusted to 2013 U.S. dollars. NPP is non-bacteremic pneumococcal pneumonia caused by *S. pneumoniae*. ‘NPP inpatient’ refers to cases of NPP that require hospitalization where as ‘NPP outpatient’ refers to cases of NPP that do not require hospitalization

^a^(Grosse et al., 2009)

^b^(Molinari et al., 2007)

^c^(Turner et al., 2006)

^d^(Weycker et al., 2012)

^e^(Tseng et al., 2011)

^f^(Yawn et al., 2007)

^g^(Singhal et al., 2011)

^h^(Masseria & Krishnarajah, 2013)

^i^(Lee et al., 2004)

Of the $26.5B total estimated cost of adult VPD in the United States among adults aged 50 and older, $15.3B (58 %) (95 % CI $11.3B, $20.9B) was attributable to adults 65 and older (Table 2b). Among adults 65 and older, influenza made up 77 % of the total annual number of cases of adult VPD and 54 % ($8.3B; 95 % CI $3.7B, $12.9B) of the total costs. Pneumococcal disease accounted for 8 % of total cases, but 25 % ($3.8B; 95 % CI $3.1B, $6.6B) of the total costs of adult VPD. Most (85 %, $3.2B) pneumococcal costs stemmed from NPP among adults 65 and older. Herpes zoster made up 11 and 20 % ($3.0B; 95 % CI $2.2B, $3.4B) of total cases and costs of adult VPD, respectively. Pertussis accounted for 4 % of total cases and 1 % ($212.5M; 95 % CI: $193.8M, $229.3M) of total costs of adult VPD (Fig. 1b). Among those 65 and older, medical costs accounted for 90 and 94 % of total influenza and pneumococcal costs, respectively, and 43 and 42 % of the total economic burden due to herpes zoster and pertussis, respectively.

Table 3 highlights the estimated economic burden of the four adult VPDs for each US state. Sensitivity analyses simulated the impact of various epidemiological scenarios on the total estimated economic burden (Table 4). Notably, a pandemic flu that resulted in 20–45 % of the adult population aged 50 and older contracting influenza in a given year could increase the total annual economic burden of the four adult VPDs by an estimated $23.6–67.9B (roughly 2–3.5 times the base case estimate) for adults 50 and older, of which $10.1–28.4B was attributable to those 65 and older (Table 4). Finally, a scenario simulating an aging population in which the number of individuals 65 and older increases by 25–50 % (as expected by 2020–2030) (Ortman et al., 2014) increased the total annual economic burden by an estimated $3.1–6.2B for those 65 and older (Table 4).

**Table 3:** Estimated total annual costs (medical + indirect costs) (in millions) caused by four major adult vaccine-preventable diseases in the United States in 2013, 2013 US dollars by state for adults aged, a 50 and Older, b 65 and Older

State/territory	Influenza (80 % medical)	Pneumococcal (91 % medical)	Herpes zoster (37 % medical)	Pertussis (42 % medical)	Total (73 % medical)
a					
United States	$16,004.6	$5064.7	$5046.9	$397.7	$26,513.8
Alabama	$254.3	$79.4	$80.5	$6.3	$420.5
Alaska	$30.4	$8.0	$8.9	$0.7	$48.0
Arizona	$339.5	$109.1	$109.4	$8.5	$566.5
Arkansas	$154.9	$49.3	$49.6	$3.9	$257.6
California	$1766.6	$554.8	$551.9	$43.9	$2917.1
Colorado	$248.1	$74.0	$76.9	$6.2	$405.0
Connecticut	$196.3	$64.2	$61.8	$4.9	$327.1
Delaware	$51.0	$16.0	$16.3	$1.3	$84.6
District of Columbia	$27.0	$8.6	$8.5	$0.7	$44.7
Florida	$1165.4	$395.0	$379.0	$29.1	$1968.4
Georgia	$451.2	$133.0	$139.2	$11.2	$734.6
Hawaii	$74.7	$24.9	$24.1	$1.9	$125.6
Idaho	$79.5	$24.5	$25.2	$2.0	$131.2
Illinois	$636.9	$202.1	$199.9	$15.8	$1054.7
Indiana	$332.0	$105.0	$104.3	$8.2	$549.6
Iowa	$166.6	$56.1	$53.5	$4.1	$280.4
Kansas	$145.2	$47.4	$46.1	$3.6	$242.3
Kentucky	$229.1	$71.0	$72.3	$5.7	$378.0
Louisiana	$226.1	$68.9	$70.5	$5.6	$371.1
Maine	$83.0	$26.3	$26.5	$2.1	$137.8
Maryland	$297.7	$92.0	$92.6	$7.4	$489.7
Massachusetts	$355.2	$115.2	$111.8	$8.8	$591.1
Michigan	$537.0	$169.9	$169.2	$13.3	$889.4
Minnesota	$277.9	$88.2	$87.1	$6.9	$460.1
Mississippi	$148.3	$45.9	$46.8	$3.7	$244.7
Missouri	$321.8	$102.6	$102.0	$8.0	$534.5
Montana	$58.4	$18.4	$18.6	$1.5	$96.8
Nebraska	$94.2	$30.9	$30.0	$2.3	$157.5
Nevada	$136.2	$41.0	$42.8	$3.4	$223.3
New Hampshire	$75.8	$23.3	$23.6	$1.9	$124.6
New Jersey	$464.6	$149.4	$145.6	$11.5	$771.1
New Mexico	$108.1	$33.6	$34.4	$2.7	$178.8
New York	$1011.6	$327.3	$319.5	$25.1	$1683.5
North Carolina	$498.2	$154.6	$157.1	$12.4	$822.4
North Dakota	$36.7	$12.3	$11.6	$0.9	$61.5
Ohio	$626.4	$201.0	$198.1	$15.6	$1041.1
Oklahoma	$193.0	$60.7	$60.9	$4.8	$319.4
Oregon	$212.6	$67.3	$68.2	$5.3	$353.4
Pennsylvania	$725.3	$240.3	$231.8	$18.0	$1215.4
Rhode Island	$57.7	$19.4	$18.4	$1.4	$96.8
South Carolina	$253.7	$77.9	$80.6	$6.3	$418.5
South Dakota	$44.2	$14.6	$14.2	$1.1	$74.1
Tennessee	$337.9	$104.1	$106.8	$8.4	$557.2
Texas	$1126.4	$338.2	$347.5	$27.9	$1840.0
Utah	$105.7	$32.3	$32.9	$2.6	$173.4
Vermont	$37.3	$11.6	$11.8	$0.9	$61.6
Virginia	$407.4	$124.5	$127.0	$10.1	$669.0
Washington	$349.6	$107.8	$109.9	$8.7	$576.0
West Virginia	$110.1	$35.1	$35.3	$2.7	$183.3
Wisconsin	$308.5	$98.9	$97.2	$7.7	$512.3
Wyoming	$29.6	$8.9	$9.2	$0.7	$48.4
b					
United States	$8312.8	$3787.1	$3017.4	$212.5	$15,329.9
Alabama	$134.3	$59.4	$48.4	$3.4	$245.6
Alaska	$12.2	$4.9	$4.3	$0.3	$21.7
Arizona	$189.6	$84.2	$68.7	$4.8	$347.3
Arkansas	$84.4	$37.6	$30.6	$2.2	$154.7
California	$892.3	$409.6	$323.9	$22.8	$1648.6
Colorado	$120.0	$52.7	$43.0	$3.1	$218.8
Connecticut	$101.3	$48.4	$37.1	$2.6	$189.4
Delaware	$27.3	$12.1	$9.9	$0.7	$50.1
District of Columbia	$13.8	$6.4	$5.0	$0.4	$25.5
Florida	$678.3	$314.1	$247.9	$17.3	$1257.6
Georgia	$221.6	$94.9	$79.2	$5.7	$401.4
Hawaii	$41.0	$19.3	$15.0	$1.0	$76.3
Idaho	$41.5	$18.2	$15.0	$1.1	$75.8
Illinois	$323.9	$150.1	$118.1	$8.3	$600.4
Indiana	$170.5	$78.2	$61.8	$4.4	$314.9
Iowa	$89.3	$43.3	$32.9	$2.3	$167.7
Kansas	$75.6	$35.9	$27.8	$1.9	$141.2
Kentucky	$118.4	$52.6	$42.8	$3.0	$216.7
Louisiana	$113.0	$50.1	$40.8	$2.9	$206.8
Maine	$43.9	$19.8	$15.9	$1.1	$80.8
Maryland	$147.8	$67.1	$53.5	$3.8	$272.1
Massachusetts	$184.3	$86.8	$67.3	$4.7	$343.1
Michigan	$276.7	$126.7	$100.5	$7.1	$511.0
Minnesota	$140.5	$65.4	$51.2	$3.6	$260.7
Mississippi	$77.1	$34.1	$27.8	$2.0	$141.0
Missouri	$169.1	$77.3	$61.6	$4.3	$312.2
Montana	$30.7	$13.8	$11.1	$0.8	$56.4
Nebraska	$49.3	$23.4	$18.0	$1.3	$92.0
Nevada	$70.9	$30.2	$25.2	$1.8	$128.1
New Hampshire	$37.8	$17.0	$13.6	$1.0	$69.4
New Jersey	$239.1	$111.9	$87.4	$6.1	$444.5
New Mexico	$57.2	$25.2	$20.6	$1.5	$104.5
New York	$526.7	$246.7	$192.3	$13.5	$979.2
North Carolina	$261.4	$115.3	$94.0	$6.7	$477.5
North Dakota	$19.1	$9.4	$7.0	$0.5	$36.0
Ohio	$326.1	$151.1	$118.8	$8.3	$604.4
Oklahoma	$102.0	$45.6	$37.0	$2.6	$187.2
Oregon	$112.5	$50.7	$40.7	$2.9	$206.7
Pennsylvania	$389.0	$184.4	$142.6	$9.9	$725.9
Rhode Island	$30.3	$14.8	$11.1	$0.8	$57.1
South Carolina	$135.0	$58.2	$48.4	$3.5	$245.0
South Dakota	$23.2	$11.1	$8.5	$0.6	$43.4
Tennessee	$176.8	$77.4	$63.6	$4.5	$322.4
Texas	$552.1	$242.8	$198.9	$14.1	$1008.0
Utah	$52.6	$23.5	$19.0	$1.3	$96.4
Vermont	$19.1	$8.5	$6.9	$0.5	$35.0
Virginia	$205.6	$91.0	$74.1	$5.3	$376.0
Washington	$177.0	$79.1	$63.9	$4.5	$324.4
West Virginia	$59.6	$26.7	$21.5	$1.5	$109.3
Wisconsin	$157.9	$73.9	$57.7	$4.0	$293.6
Wyoming	$14.4	$6.4	$5.2	$0.4	$26.4

**Table 4:** Sensitivity analyses estimating changes in total annual cost (in millions) of four major adult vaccine-preventable diseases in 2013 based on hypothetical epidemiological scenarios, 2013 US dollars

	Adults aged 50 and older		Adults aged 65 and older	
	Total cost (millions)	$ Change^a^ (millions)	Total cost (millions)	$ Change^a^ (millions)
Base case	$26,513.8	*n.a.*	$15,329.9	*n.a.*
% Of US population that develops influenza annually				
5	$20,993.9	−$5519.9	$11,659.4	−$3670.5
20	$50,110.5	$23,596.7	$25,423.7	$10,093.8
25	$62,972.9	$36,459.1	$30,011.9	$14,682.0
40	$94,457.3	$67,943.5	$43,754.6	$28,424.7
% Of patients who develop pneumonia who are hospitalized				
30	$25,876.8	−$637.0	$14,473.2	−$856.7
50	$27,684.7	$1170.9	$15,791.7	$461.8
60	$28,590.5	$2076.7	$16,450.9	$1121.0
% Of herpes zoster patients who develop PHN				
10	$26,106.2	−$407.6	$15,044.5	−$285.4
20	$26,638.0	$124.2	$15,428.7	$98.8
30	$26,886.4	$372.6	$15,667.6	$337.7
40	$27,234.3	$720.5	$15,931.1	$601.2
% Increase in population aged ≥50 and ≥65 years				
+25	$31,887.8	$5374.0	$18,407.4	$3077.5
+50	$37,261.6	$10,747.8	$21,485.0	$6155.1
+100	$48,009.4	$21,495.6	$27,640.0	$12,310.1

^a^Absolute dollar amount change (in millions) from the base case estimate

*n.a.* is not applicable, *PHN* is post-herpetic neuralgia

## Discussion

Results from the epidemiological model suggested that the economic impact of four adult VPDs (influenza, pneumococcal disease, herpes zoster, and pertussis) is considerable, and that in 2013, the economic impact attributable to adult VPD for US adults aged 50 and older was $26.5B ($15.3B for those 65 and older). This economic burden would be even greater if mortality costs (i.e., the value of future income lost by premature death) and leisure time costs (i.e., the value of time spent when not working forgone by illness) were included in the analyses. Moreover, results of the model showed that although influenza (often the focus of most adult immunization programs) accounted for the majority of cases of adult VPD (81 % of adults 50 and older and 77 % of adults 65 and older), from a cost perspective, pneumococcal disease and herpes zoster both represent a significant economic burden in addition to influenza. Specifically, among adults 65 and older, although pneumococcal disease represented only 8 % of total cases, it contributed to one-fourth of the total costs, with the large majority (85 %) of pneumococcal burden due to NPP. Likewise, herpes zoster made up 11 % of total cases, but 20 % of total adult VPD costs among those 65 and older. Thus, broadening adult immunization efforts beyond influenza only may help reduce the economic burden of disease.

Based on this model and consistent with previous research (Maciosek et al., 2006a, b), a pneumococcal vaccination effort, primarily focused on reducing the burden of NPP, may be a logical place to start—especially if reducing medical costs is the primary goal. Recent data from a large randomized controlled trial suggested that PCV13 is effective for preventing vaccine-type pneumococcal community-acquired pneumonia in older adults (Bonten et al., 2015; Tomczyk et al., 2014). Subsequently, in September 2014, ACIP recommended PCV13 (in addition to PPSV23) for routine use in all adults 65 and older, estimating that “…10 % of community-acquired pneumonia cases in adults aged ≥65 years are caused by PCV13 serotypes and are potentially preventable with the use of PCV13 in this population” (Tomczyk et al., 2014).

Adult uptake of age-appropriate vaccination, however, remains low for most vaccines routinely recommended for adults (CDC, 2010, 2013a, 2014; Williams et al., 2014). Thus, it is critical to devise successful adult immunization implementation plans to improve the immunization coverage rate for these adult VPDs. Research about vaccination has suggested that barriers exist at the patient, provider, and system level. The most salient patient barriers include lack of awareness of the need for the vaccine, lack of recommendation by a healthcare provider, fear of side effects, busy schedules and competing demands, and lack of belief that receiving the vaccine is ‘wise’ (Lau et al., 2012; Zimmerman et al., 2003). Education may also play an important role, as prior studies have documented a relationship between low education status and poor vaccination rates (CDC, 2002). Yet, previous research has shown that—even among elderly, low-literate, low education level, indigent populations—a simple, low-literacy educational tool can increase pneumococcal vaccination rates and encourage patient-physician discussions about pneumococcal vaccination (Jacobson et al., 1999). In addition, previous studies have demonstrated that patients’ attitudes about vaccination are also important (Nowalk et al., 2004; Zimmerman et al., 2003a, b). While traditional health education models have focused on knowledge alone, research suggests that both patients’ knowledge (e.g., about benefits and risks) and attitudes (i.e., how a person feels about vaccination) are important to understanding vaccination uptake. Specifically, for individuals who are firmly fixed on refusing vaccination, research has shown that they are often still open to listening to or even complying with healthcare provider recommendations (Nichol et al., 1996). Understanding the nature of the barrier is key to defining the appropriate strategy to overcome it.

The second type of barrier centers on provider issues. Research has found that some physicians do not believe vaccines are efficacious and thus do not recommend vaccination to their patients, do not stock the vaccines, and fail to act as champions for adult vaccination (Kimura et al., 2007; Nichol & Zimmerman, 2001; Silverman et al., 2004). Because providers are largely responsible for educating patients, it is imperative that they have a high level of understanding about vaccination and the effect of their recommendations on patient behavior. CDC has identified four key components for reducing provider barriers: (1) assessment (measuring immunization rates), (2) feedback (inform providers about immunization performance), (3) incentives (adequate reimbursement to support efforts to raise immunization rates), and (4) exchange of information (benchmarking and sharing what works) (CDC, 2012b). Additionally, in the United States, many adult patients only see specialty physicians; however, most specialists do not assume primary care responsibility for elderly patients—leaving a gap in care for many adult patients pertaining to recommending and administering immunizations (Rosenblatt et al., 1998). Thus, additional opportunities (e.g., pharmacies and the workplace) for prompting adults about the importance of immunization and administering vaccines should be explored.

The third area where barriers often exist is at the health care system-level (Nichol & Zimmerman, 2001). System-level barriers include poor vaccination refrigerator location, having a central pharmacy and not stocking the vaccines at individual clinics, and lack of reminder and tracking systems. These are relatively simple solutions that can have big impacts on vaccination uptake. Specifically, a previous review demonstrated that patient reminder systems were effective in improving immunization rates nearly 80 % of the time, irrespective of baseline immunization rates, patient age, setting, or vaccine type (Szilagyi et al., 2000). Other meta-analyses have consistently demonstrated that system-oriented strategies can increase immunization rates by 20–40 % (Gyorkos et al., 1994). Examples of system-level change include shared roles between the nurse and the physician, where the nurse can vaccinate according to standing orders, and where there is routine screening of vaccination status, which could be part of the vital signs protocol (Lau et al., 2012). In addition, previous research has demonstrated that tailored telephone reminders about pneumococcal vaccination can double the odds of a patient being vaccinated in a diverse managed care setting (Winston et al., 2007). Tracking vaccination history is also key to implementing timely and accurate reminder and recall programs. Immunization information systems (IIS)—confidential registries that consolidate immunizations records from all health care encounters (e.g., pharmacies, retail clinics, primary care providers, subspecialists, hospitals, employers, and public health departments) into one centralized repository—have been widely utilized for children with notable success in improving immunization rates (Groom et al., 2014). IIS, however, remain underutilized in adults, primarily because adults are vaccinated by multiple and diverse health care providers in a variety of health care settings (CDC, 2013b). Recent reports suggest that IIS could be used in adults for point-of-care support and overall population health management, and that IIS utility for tracking and consolidating adult immunization records should be continually improved and evaluated (CDC, 2013b). The Community Preventive Services Task Force recommends both reminder and recall interventions and IIS utilization based on strong evidence of effectiveness in improving vaccination coverage (The Community Preventive Services Task Force, 2008, 2014).

The Veterans Administration (VA) has been a paradigm of success for immunizing adults (Nichol, 1992, 1998). Historically, the VA has used a multidisciplinary, multimodal approach that includes reminders and standing orders, which empowers nurses to vaccinate per protocol, and freestanding express immunization clinics so that individuals can come and go quickly at their leisure. The VA actively monitors immunization rates as part of their quality measures and provides clinicians with feedback about how well they are vaccinating individually and as an organization. Some VA sites employ a dedicated prevention nurse who sees the patients before the physician does, with the primary goal of identifying all needed preventive services. The VA utilizes an electronic medical record system to track vaccination history and prompt health care providers if immunizations are needed (Nichol, 1992, 1998).

This epidemiological model is not without limitations. The model relied on the human capital methodology of calculating cost, which does not tend to capture the monetary or nonmonetary value of pain, suffering, and premature death due to disease. In 2013, it was estimated that approximately 54,000 individuals aged 50 and older died from influenza and pneumonia. The large majority of which (>48,000;~90 %) occurred among adults 65 and older (Murphy et al., 2013). From a societal perspective, this premature death—which we did not capture in this analysis—has an economic value. As such, the output of this model underestimated indirect costs relative to other cost calculation methods. It is also important to point out that the methodology and output of the model reflect only the burden of disease attributable to the four adult VPDs included in the analysis. Because no vaccine is 100 % effective, nor does it cover all disease strains or serotypes, this model does not reflect the actual amount of disease that would necessarily be prevented with vaccination or by improving vaccination coverage. Instead, the model should be interpreted as the US burden of disease in 2013, among both vaccinated and unvaccinated persons, that is attributable to each infectious disease included in the model. For example, influenza vaccines do not prevent disease from all influenza strains, and pneumococcal vaccines prevent only pneumococcal disease caused by serotypes included in the vaccine. However, these vaccines are designed to cover the most prevalent strains and serotypes that typically cause disease. Moreover, although the model does age-adjust incidence rates and diseases costs where possible for differences in state population demographics, it does not account for differences in other characteristics (e.g., race/ethnicity, health care access, population density, rural/urban mix, immunization rates) that may affect overall incidence rates or costs from one state to another. Finally, although the model did include four major adult VPDs, other adult VPDs (e.g., hepatitis) were not included and should be examined in the future.

In spite of these limitations, this model gives a broad overview of the societal cost attributable to four major adult VPDs for the United States as a whole and for each state. Although modeling of this type is not an exact science, the model uses peer-reviewed inputs for age-adjusted, disease-specific incidence and cost, and provides an educated ‘best guess,’ with sensitivity analyses, as to what the burden of disease due to adult VPD was in the United States in 2013.

Childhood immunization programs in the United States have nearly eliminated diseases that once claimed the lives of countless children. Adult immunization programs, however, have been slow to evolve. Many reasons have been identified for the nation’s poor adult immunization rates. These include: variable insurance coverage among patients, a lack of incentives for health care providers to stock and deliver adult immunizations, a lack of a ‘medical home’ for adult patients, the absence of reminder-recall and vaccination history tracking systems, insufficient development of standing order programs for vaccination, and lack of assessment of practice-level vaccination rates with feedback to staff members in medical practices (Lau et al., 2012).

In order for adult immunization efforts to increase, the value of preventing these diseases, in both human and economic terms, must be recognized. Results from this analysis provide an estimate of the cost attributable to four major adult VPDs, and highlights the importance of addressing adult vaccination uptake. Sensitivity analyses suggested that as the US population ages over the next decade and beyond (Ortman et al., 2014), without increased prevention efforts, these costs will dramatically increase. Previous studies have suggested that system-wide changes, especially the implementation of standing orders for vaccination, assigning non-physician personnel vaccination responsibilities, and in-person clinician recommendation have the greatest impact on increasing uptake (Lau et al., 2012). Implementing these types of interventions in subpopulations most at risk for developing adult VPDs and at greatest risk for not being vaccinated will likely yield the greatest benefit. Results from this model make it evident that a fundamental shift in the culture within which vaccines (and indeed preventive care as a whole) are provided to adults is required, and this analysis should provide a stimulus for policy makers to undertake this ambitious goal. Failure to do so, however, based on this model and previous reports, will continue to cost the United States billions of dollars each year.
